# Sudden death in a patient with epilepsy and arterial hypertension: time for re-assessment

**DOI:** 10.6061/clinics/2021/e3023

**Published:** 2021-05-27

**Authors:** Fulvio A. Scorza, Antonio Carlos G. de Almeida, Carla A. Scorza, Josef Finsterer

**Affiliations:** I Disciplina de Neurociencia, Escola Paulista de Medicina/Universidade Federal de Sao Paulo (EPM/UNIFESP), Sao Paulo, SP, BR.; II Centro de Neurociencias e Saude da Mulher “Professor Geraldo Rodrigues de Lima”, Escola Paulista de Medicina/Universidade Federal de Sao Paulo (EPM/UNIFESP), Sao Paulo, SP, BR.; III Laboratorio de Neurociencia Experimental e Computacional, Departamento de Engenharia de Biossistemas, Universidade Federal de Sao Joao del-Rei (UFSJ), Sao Joao del-Rei, MG, BR.; IV Krankenanstalt Rudolfstiftung, Messerli Institute, Vienna, Austria.

Epilepsy is a common severe neurological disease independent of age, race, social class, geographic, or national boundaries (1). Refractory epilepsy patients require the most time, attention, effort, and focus from neurologists due to the frequency and severity of their seizures. Refractory epilepsy is a major cause of disability, comorbidity, stigma, costs, and mortality (2-5). Epilepsy has been associated with an increased risk of premature death, particularly among refractory epilepsy patients (2,5,6). Sudden Unexpected Death in Epilepsy (SUDEP) is the most important direct epilepsy-related cause of death, accounting for 10-50% of all deaths (2,5,6). SUDEP is defined as “the sudden, unexpected, witnessed or unwitnessed, non-traumatic, and non-drowning death in patients with epilepsy, with or without evidence for a seizure, and excluding documented status epilepticus, in which postmortem examination does not reveal a toxicological or pathoanatomical cause of death” (7). The incidence of SUDEP is approximately 1 in 4,500 children per year and 1 in 1,000 adults per year (2,8-10). The risk factors and predictors for SUDEP include early adulthood, early onset of epilepsy, long duration of epilepsy, a high number of anti-epileptic drugs (AEDs), and cold temperature (5,9,11,12). Several studies have indicated that the frequency of nocturnal generalized tonic-clonic seizures was the leading clinical risk factor for SUDEP (2,6,13-17). Structural and functional heart changes were documented in epilepsy patients, suggesting the involvement of cardiac arrhythmias and autonomic dysfunction in SUDEP (6,10,12,13,16-20). Moreover, patients with drug-resistant epilepsy were found to have an abnormal resting autonomic function, including reduced heart rate variability, baroreflex sensitivity, and electrodermal activity (19). Seizure control is still the most effective management for SUDEP (2,14,15,21-23). Possible preventive strategies include stress reduction, engaging in physical activity and sports, dietary management (e.g., omega-3 supplementation), supervision at night, and living with a dog (2,14,15,21-23). Due to the complex nature of SUDEP, early identification of the clinical risk factors has remained challenging. Thus, it is important to investigate the pathophysiology of arterial hypertension (AHT) and its implications on SUDEP.

AHT has recently been related to the risk of sudden cardiac death (24). This also applies to epilepsy patients (25). Studies on the mechanism behind AHT and the development of cardiovascular abnormalities and sudden death have inspired studies on the risk of SUDEP in epilepsy patients (26). Epilepsy and AHT are common chronic diseases that can coexist in the same individual (24). The relationship between refractory epilepsy, AHT, and sudden death was observed in the following case. A 42-year-old man with normal neurodevelopment experienced a severe febrile seizure during the first year of life. When he was 11 years old, the patient started having focal or bilateral tonic-clonic seizures. On interictal electroencephalography, epileptiform discharges were recorded in the left anterior temporal lobe projection. In addition, brain magnetic resonance imaging showed left mesial temporal sclerosis (loss of internal architecture of hippocampus and reduced hippocampal volume). Several AED therapies failed to control his seizures, and the patient developed refractory epilepsy. He was also diagnosed with AHT during a routine clinical visit. He was referred to a cardiologist and was prescribed medications, but he did not adhere to his antihypertension treatment (mainly driven by forgetfulness to take medications). Thus, his blood pressure remained poorly controlled. The patient was found dead one Sunday morning. The patient did not have other comorbidities aside from AHT and epilepsy. Postmortem examination was not carried out. His death was clinically classified under probable SUDEP (27).

This case and previous related studies have emphasized several learning points. AHT was involved in the pathophysiology and development of seizures and epilepsy (25). Furthermore, AHT was a predictor of late-onset epilepsy, regardless of vascular damage. It also indirectly promotes cerebrovascular disease, which increases the risk for acute symptomatic seizures or chronic epilepsy (25). Moreover, the coexistence of AHT and epilepsy exposes patients to multiple pharmacological treatments (25). The drug-drug interactions and mechanisms of actions should be considered in AHT patients taking multiple drugs (25). Epilepsy and AHT are common chronic diseases with severe implications for public health (25,28), and patients with AHT and epilepsy have an increased mortality rate and risk of sudden death.

Isolated AHT and elevated pulse pressure are involved in the development of brain complications (29). Thus, AHT increases the risk not only for cerebrovascular morbidity and mortality, but also for cognitive impairment and dementia (29). Since AHT increases the risk of SUDEP, it should be considered a risk factor for developing fatal events in epilepsy. Finally, a task force should be established to assess the state of knowledge on this issue and identify clinical gaps in the diagnosis and treatment of this high‐risk population.
